# The (inter)visual politics of border security: Co-constituting gender and race through Frontex’s Risk Analysis

**DOI:** 10.1177/09670106231182314

**Published:** 2023-08-03

**Authors:** Columba Achilleos-Sarll, Julia Sachseder, Saskia Stachowitsch

**Affiliations:** University of Birmingham, UK; Central European University, Austria; Central European University, Austria

**Keywords:** Border security, Frontex, intersectionality, intervisuality, postcolonial feminism, visual politics

## Abstract

Visuals, including photographs and data visualizations, play a crucial role in the politics of EU border security, both as an internal governance tool (e.g. in surveillance) and as an external means of communication/representation (e.g. in photojournalism). Combining scholarship on photographic representations of migration with literature on surveillance technologies and data visualizations, we argue that these visuals interact to reproduce gendered and racialized meanings of migration and border security. Using a feminist postcolonial lens, we develop an intervisual framework for studying how processes of gendering and racialization render subjects, practices and spaces knowable at the intersection between these visuals. We apply this framework to a case study of Frontex’s Risk Analysis Reports (2010–2021) and demonstrate how it is applicable to other security institutions. The intervisual analysis reveals how the migrant Other and (white) European are visually reproduced through: 1) the (in)visibilization of bodies; 2) the ascription and denial of agency; and 3) the spatialization of borders as ‘frontier imaginings’ that oscillate between fortification and expansionism. The intersectional co-constitution of gender and race, we conclude, is central to the visual politics of Frontex, contributing to problematizing migrants and migration and legitimizing violent border practices.

## Introduction

Visuals are a powerful tool in the representation, communication and governance of migration and the ‘European migrant crisis’. Across news media coverage, photographs such as those of Alan Kurdi, the body bags washed up at Lampedusa and overcrowded migrant boats have taken on an iconic status (Adler-Nissen et al., 2019). However, photographs are only one medium through which the ‘migrant crisis’ and migration are visually represented and reproduced. EU institutions, such as the European Border and Coast Guard Agency Frontex, which is responsible for managing the EU’s external borders, produce material that includes photographs alongside data visualizations, such as maps, charts and graphs. Drawing on gendered and racialized stereotypes and hierarchies, both types of visuals contribute to problematizing migration and justifying the EU’s repressive border security practices. We argue that part of what renders migrants and migration knowable and consequently governable emerges at the intersection between these visual modes that together construct (non-)European bodies, practices and spaces in hierarchically gendered and racialized ways. Developing a feminist postcolonial framework that draws on the concept of intervisuality (Mirzoeff, 2007), this article is the first to examine the visual politics of Frontex by attending to the interaction between different visual genres, how this interaction renders migrants and migration knowable, manageable and thus governable, and how they are shaped by, and shape, the co-constitution of gender and race.

The central actor operating at the EU’s external borders, Frontex is responsible for the prevention and detection of ‘irregular’ migration and cross-border crime, surveillance of migratory flows and Search and Rescue operations (SAR), among other tasks. The agency, which has grown exponentially through two mandate extensions in 2016 and 2019 (Fjørtoft, 2020; Perkowski, 2021), produces and publishes a range of visual materials indicating how the EU ‘sees’ migrants and migration (Silberstein, 2021). Like all visuals, this material does not simply represent an objective ‘reality’ but co-constitutes that reality (Barthes, 1977 [1964]; Sontag, 1977) by weaving together a series of representations about subjects/objects that renders them meaningful. Rather than an innocent reflection of ‘reality’, visuals are ‘socially constructed and culturally located’ (Welland, 2017: 528), bound within ‘conditions of visibility’ (Pidduck, 2011: 12). Visuals, like all modes of communication, help create the conditions upon which a phenomenon, such as migration, ‘can be known and acted upon’ (Doty, 1996: 6), making some border security policies and practices appear logical and even desirable, while foreclosing others.

Risk analysis, the central knowledge and surveillance practice through which Frontex manages migration, is illustrative of how visuals become consequential in EU border politics. This practice utilizes visuals for two main purposes: 1) internal governance; and 2) external communication, including public relations. Data visualizations, particularly migratory maps, are central to governance practices as they determine when migration becomes a ‘risk’ to the EU’s external borders, and therefore when and how Frontex should intervene through, for example, tightening border controls and enhancing surveillance. Additionally, visuals serve as a means of representation and communication that conveys the agency’s activities to EU policymakers, member state representatives and the general public. Across both functionalities, visuals form part of Frontex’s knowledge practices and operations that are constitutive of its heavily criticized security and border management activities, including illegal pushbacks (Davies et al., 2022).

Against this backdrop, Frontex’s annual Risk Analysis Reports (RARs), the primary output of the agency and its knowledge production, are concerned with monitoring and governing migration. RARs provide a unique set of empirical material for studying the interaction between photographs and data visualizations, how they function as both forms of representation and governance, and how they are implicated in the gendered and racialized logics of EU border security. While existing scholarship on EU migration governance demonstrates how gender and race determines who is ‘deserving’ of asylum and protection (e.g. Bird 2022; Welfens and Bonjour, 2020), and the literature specifically on Frontex examines how the language and discourse of risk analysis is constituted through gender and race (Sachseder et al., 2022; Stachowitsch and Sachseder, 2019), this study offers the first systematic intervisual analysis of RARs. We thus ask how the visual environment of risk analysis, and of Frontex more generally, contributes to the reproduction of gendered and racialized hierarchies, stereotypes and exclusions.

To examine how gender and race are intervisually co-constituted in risk analysis, we draw on two bodies of literature. The first is concerned with how migration is represented in photojournalism (e.g. Amoore, 2007; Bleiker et al, 2013; Burrell and Hörschelmann, 2019; Chouliaraki and Zaborowski, 2017; Hansen et al., 2021; Musarò 2017), and the second focuses on the interplay between migration surveillance and mapping technologies that feed data visualizations (e.g. Bellanova et al., 2021; Leese et al., 2021; Rothe et al., 2021; Tazzioli and Walters, 2016; Van Houtum and Bueno Lacy, 2020). Using a feminist postcolonial lens that draws attention to the co-constitution of gender and race in visualizations of threat, risk and (in)security, we develop an analytical framework that examines how photographs and data visualizations together construct gendered and racialized meanings *intervisually* (Mirzoeff, 2001, 2007) and *intersectionally* (Crenshaw, 1989; Mohanty 1984).

We apply this framework to the visuals published in Frontex’s RARs (2010–2021) with a focus on the representation of subjects, practices and spaces. We also consider how these representations change and/or persist over time in the wider context of Frontex’s enormous expansion and growth since 2016. We find that the interaction between different images in RARs intervisually reproduces the racialized migrant and white European in three ways: first, through the (in)visibilization of bodies; second, through ascribing or denying agency to migrants and border guards; and third, through the spatialization of borders as ‘frontier imaginings’ that oscillate between colonial themes of fortification and expansionism. These findings demonstrate how the visual politics of Frontex encode processes of Othering that target those at the bottom in terms of surveillance, exclusion and violence thus legitimizing the violent structures of the EU’s border security machinery. Our framework is applicable beyond Frontex and could be used to study how other border and/or security actors, such as the International Organization for Migration (IOM), the EU in the context of the Common Security and Defence Policy (CSDP) or NATO, who reproduce gendered and racialized hierarchies by drawing from different visual genres at the intersection between representation and governance.

## The visuality–migration nexus

The scholarship on gender, race, EU migration governance and border security not only highlights how migration policies and security practices impact women and other marginalized and racialized groups (e.g. Bird, 2022; Freedman et al., 2022), but it also demonstrates how gender and race are co-constitutive of conceptualizations of (in)security. This includes constructions of migrants as either passive victims ‘at risk’ or ‘risky’ in contrast with the EU as a legitimate border security actor (e.g. Aradau, 2004; Gray and Franck, 2019; Pallister-Wilkins, 2015, 2021) as well as notions of un/deservingness that determine entitlement to protection and asylum (Welfens and Bonjour, 2020). Turning to Frontex specifically, Sachseder et al. (2022; Stachowitsch and Sachseder, 2019) demonstrate that gender and race inform understandings of both risk and crisis that help legitimize the agency’s practices and establish the rationale that fuels institutional growth. We contribute to this scholarship by focusing on how the agency’s visual environment produces and transforms gendered and racialized hierarchies that make borders, migration and migrants known/knowable and thereby governable.

To make sense of the interlinked visual functionalities of risk analysis, our research further draws on insights from two bodies of scholarship that are constitutive of the ongoing conversation around visuality, migration and border security to which this article contributes. The first examines the construction of migrants and migration in photojournalism (e.g. Adler-Nissen et al., 2020; Berents, 2019; Bleiker et al., 2013; Chouliaraki and Georgiou, 2022; Chouliaraki and Stolić, 2019; Hansen et al, 2021). This literature examines how visuals, mostly photographs, are used to communicate particular representations of migration, in particular by rendering migration a ‘crisis’. Analyzing and directing attention to several recurring motifs, such as the overflowing migrant boat, this scholarship demonstrates how visuals racialize, feminize and (hyper)masculinize the migrant as either dangerous/risky or in need of protection. Gender and race have thus been found to be important power relations informing what/who is being represented or not in the roles of threat, victim or savior (Burrell and Hörschelmann, 2019; Chouliaraki and Zaborowski, 2017; Kędra and Sommier, 2018; Saric, 2019). However, this literature focuses on how migration is communicated to the wider public rather than how security institutions themselves use visuals for governance and/or representational purposes that may increase the precarity of migrants.

We therefore draw on a second strand of literature that unpacks the use of data-driven security technologies in EU border and migration governance, including surveillance-mapping systems. These systems produce data visualizations that are productive of heterogeneous and overlapping forms of visibility and visuality (e.g. Bellanova et al., 2021; Madörin, 2020; Rothe et al., 2021; Tazzioli, 2018; Tazzioli and Walters, 2016). Particularly prominent among these data-driven technologies and visualizations are migratory maps (Van Houtum and Bueno Lacy, 2020), which depict and manage ‘crises’ from afar by monitoring migratory movements effectively out of sight (Madörin, 2020: Rothe et al., 2021). These are exemplified by Tazzioli and Walters (2016) in their analysis of the two central information exchange and foresight systems devised and operated by Frontex, the European Border Surveillance System (EUROSUR) and the Joint Operations Reporting Application (JORA). The migratory maps produced via these systems cannot be reduced to technical tools that reflect the objective collection, monitoring and reporting of migrant/migration data. Rather, they have been interpreted as a crafting of future risk scenarios according to their governability. Additionally, Madörin (2020) conceptualizes the intertwinement between the visual/scopic, digital/algorithmic and image/code as the ‘postvisual’, arguing that they work together to generate a ‘view from above [. . .] constructed through data [and] data-generating “vision machines”’ (2020: 700), producing a ‘regime of visibility’ (Tazzioli, 2018: 277). The interaction between image and code feeds into multiple and dispersed (though not necessarily unidirectional) ways of ‘seeing’ and ‘being seen’ (Madörin, 2020), which this literature argues provides the rationale for governing migration in the first place. An important part of this literature demonstrates how gender and race matter in these algorithmic systems and associated (visual) technologies by transforming ‘code’ into patterns and routes through racial, ethnic and gender profiling (Bellanova et al., 2021; Chouliaraki and Georgiou, 2022; De Genova, 2013). This can be linked to broader processes of European racial securitization that gathers data as a form of knowledge extraction akin to surveillance practices, such as fingerprinting, that were integral to the management of the colonies (Axster et al., 2021; Madörin, 2020; Massari 2021).

Notwithstanding that hierarchical constructions relating to the co-constitution of gender and race are important empirical findings in both literatures, seldom are these categories employed as either analytical lenses or constructions that are rooted in colonialism and perpetuated through postcolonial power relations (Mirzoeff, 2023; Wynter, 1996, 2003) between the EU and its ‘Other’. Furthermore, while the above literature provides valuable insights about the intersection between visibility, visuality and migration, a comprehensive study of the visual environment of Frontex that draws on photographs *and* data visualizations is currently lacking. We therefore study Frontex’s risk analysis as an important example that demonstrates how migration is both represented and governed through the visual politics of gender and race. To that end, the following section advances a theoretical and methodological framework that develops the concept of intervisuality. With this framework, we study how different visual genres in RARs interact in ways that reproduce and stabilize gendered and racialized meanings that constitute, rather than causally determine (Hansen, 2011), EU border security practices.

## A feminist postcolonial approach to intervisuality

Intervisuality is a concept regularly cited in the visual politics scholarship to describe genealogical visual codes and associated discourses as well as the process of analyzing multiple and/or diverse visual genres together (e.g. Callahan, 2020; Hall et al., 2013; Hansen, 2011; Mirzoeff 2001, 2007; Spens, 2018). Intervisuality acknowledges ‘the accumulation of meanings across different texts, where one image refers to another, or has its meaning altered by being “read” in the context of other images’ (Hall et al., 2013: 222). Through ‘interacting and interdependent modes of visuality’ (Mirzoeff, 2007: 7), meanings are reproduced at the intersection between ‘texts’ (written, spoken and visual) as well as between ‘text’ and reader/audience (Appadurai and Breckenridge, 2002). Furthermore, an intervisual reading helps uncover dominant or hegemonic visual narratives reproduced across multiple images/texts. This means that the polysemic nature of images – that is, that they elicit multiple interpretations – becomes ‘stabilized’ when different images in the same ‘visual system’ reproduce common motifs. However, while intervisuality is often used to describe a visual environment that draws on several visuals (either the same or different genres), it is rarely employed as a distinct methodology (Spens, 2018). Additionally, intervisuality has not yet been used to unpack how security actors in particular draw on multiple visual genres as a ‘vector of power’ that reproduces migration through gendered and racialized power relations (Achilleos-Sarll, 2020: 1644). We therefore develop intervisuality as a methodological approach for studying how the interplay between different visual genres operates to sustain gendered and racialized power in the context of postcoloniality.

Our approach is intersectional in that we are interested in how gender is co-constituted with other categories of difference and structures of inequality, particularly race (Crenshaw, 1989; Yuval-Davis, 2006). As racial orders are central to the field of security (among others, Achilleos-Sarll, 2023; Machold and Charrett, 2021), this approach foregrounds how gender intersects with race to construct hierarchical differences based on perceived dichotomies between masculine/feminine, white/non-white, European/non-European, rational/emotional, civilized/barbarian, us/them, among other pairings (McCall, 2005; McClintock, 1995; Mohanty, 1984; Peterson, 2010). As such, we understand race not only in terms of Othering, but also as constituting the Self through whiteness. From an intersectional perspective, we centre the *work* that processes of gendering (Zalewski, 2010) and racialization do (Machold and Charrett, 2021) to not only visually construct the ‘ideal’ Self and ‘deviant’ Other, but to generate particular ways of seeing, such as the ‘male gaze’ or ‘white sight’ (Mirzoeff, 2023), that privilege and normalize certain positions of power.

Our combined intervisual–intersectional framework starts from the premise that gender and race are formed through colonialism and reproduced through postcolonial power relations and whiteness, and asks how these have shaped and continue to shape visual practices, politics and culture (Mirzoeff, 2023; Wynter, 1996). This is particularly relevant for theorizing intervisuality in EU migration governance and border security, considering that the EU’s repressive bordering practices are a dis/continuation of control over formerly colonized lands and peoples (Hansen, 2002; Jensen, 2020; Kinnvall, 2016; Walia, 2013). Colonial patterns of meaning-making therefore structure (visual) representations in EU border security, particularly around the construction of subjects, practices and spaces. These representations rely on an active/passive binary between the European ‘subject’ and the non-European ‘object’ (Chouliaraki and Stolić, 2019), reinforcing the agency of the former as privileged interpreters and knowledge-producers of non-European lifeworlds, while devaluing the migrant Other by criminalizing or denying their agency altogether.

Self/Other subjectivities represented through differential ascriptions of agency are co-constituted with and made meaningful against the backdrop of spatial imaginings of un/inhabitable territories that need to be either protected or conquered, surveilled or controlled (Wynter, 1996: 21). These colonial imaginings not only represent far-flung territories as a racialized threat, but also constitute ‘white cartographic subjectivities’ (King, 2019: 84) by creating interior zones of safety associated with the logos against outside ‘zones of (embodied) affectability’ (2019: 87). To make sense of how colonial imaginings shape the spatial politics of EU bordering, Walters’s concept of the ‘frontier’ as moveable and outward looking is useful. As a political ambition rather than a fully realized goal (2004: 679), the spatial imagining of the ‘frontier’ is historically linked to North American settler colonialism (Turner, 1920) and constructs borders as spaces of ambiguity, danger but also potential (Imamura, 2015: 97). The frontier is a ‘meeting point between savagery and civilization [. . .] between a power and its outside [. . .] a space of interaction, assimilation, violence, and also pacification’ (Walters, 2004: 687). It provides the backdrop not only for territorial rearrangements but also for identity formation of the white ‘pioneer’ through the physical subjugation of racialized bodies and feminized spaces, as well as through knowledge practices such as surveillance and mapping that were used to manage the colonies (Manchanda, 2020). As regional blocs such as the EU ‘are also acquiring frontier characteristics’ (Walters, 2004: 674), these ‘frontier imaginings’ (Prout and Howitt, 2009) also become formative of EUropean identity. Visual representations of territories and borders, both in cartography and photography, are therefore indicative of how gender and race are inscribed into and reproduced through colonial imaginings of non-/EU space and the frontiers between them.

Using our intervisual-intersectional framework, we examine processes of visual gendering and racialization of different subjects, such as migrants and border guards; depicted practices, such as border patrolling, surveillance or SAR, and the agency they ascribe and/or deny; as well as spatial imaginations of the border and (non-)EU spaces. To unpack how these constructions are formed at the intersection of different visual modes, the following section details our case selection and introduces the materials and methods of analysis.

## Case selection, materials and methods

Founded in 2004, Frontex is considered the central institution managing migration and securing the EU’s external borders through operational and technical assistance to member states and through the harmonization of EU border management (Boswell and Hampshire, 2017; Neal, 2009; Perkowski, 2021). Two mandate reforms in 2016 and 2019 dramatically increased personnel and financial resources as well as strengthened the agency’s role in the implementation of the EU’s approach to Integrated Border Management (IBM). This widened its capabilities and tasks, particularly its activities in/with third countries in the context of increased border externalization, including its surveillance capabilities through the integration of EUROSUR and the European Travel Information and Authorisation System (ETIAS). As a consequence of its institutional power and growth, Frontex has gained a hegemonic role in the representation, governance and treatment of migrants at the EU’s borders (Perkowski, 2021). Visuality is a constitutive part of these processes, and can be understood both as a ‘technical’ tool in the surveillance and datafication of migrants/migration, and as a ‘political’ tool in the representation and problematization of migrants/migration to a wider EU audience. Frontex is therefore a particularly important case study for understanding the visual politics of EU migration and border politics.

Central to Frontex’s activities is risk analysis. Risk analysis collects and disseminates data about migratory movements towards the EU to assess the risk of ‘illegal’ or ‘irregular’ cross-border activities (Paul, 2017). It determines Frontex’s operations, but also informs wider EU policymaking, shaping the relationship between EU institutions and member states (Fjørtoft, 2020). Yet, rather than simply a technical and operational practice through which migration is overseen, risk analysis is a deeply political set of hegemonic knowledges (Gundhus, 2018) and thus a ‘form of power’ (Horii, 2016: 242). Photographs and data visualizations matter at different stages in the risk analysis process, and are prominent within its central risk analysis publication, the RARs, which provide the empirical material for our visual analysis. Data visualizations, such as migratory maps, graphs and charts are compiled from data collected at the border through the aforementioned surveillance systems EUROSUR and JORA which utilize technologies such as drones, satellites and offshore sensors. These systems function as governance tools and foresight systems to judge whether migratory movements are ‘risky’ and therefore whether to intervene by assisting national border authorities (Tazzioli, 2018). Additionally, risk analysis draws heavily on photographs as a means to represent and justify its activities to various audiences through projections of risk, threat and border vulnerabilities. The practice of risk analysis is (in part) made meaningful through visuality, which is our methodological entry point to investigate how border security is intervisually co-constituted by, and constitutive of, gendered and racialized hierarchies.

Our dataset consists of all visual material in the annual RARs published between 2010 and 2021, which are publicly available on the agency’s website. This includes all photographs (187) and data visualizations (220). Maps are central in our analysis because they are the most common data visualization included in RARs and because of their essential role in spatializing hierarchies and (global) inequalities (King, 2019). Over time, we observe that RARs have become more comprehensive in their style and substance, with both the quantity and quality of visuals increasing. The 2010 RAR (Frontex, 2010), for example, includes only two rather basic maps and three unprofessional-looking photographs, whereas the 2021 RAR (Frontex, 2021) features 18 glossy photographs and 26 data visualizations, including eight maps.

According to the copyright information, the photographs are attributed mainly to Frontex but some are sourced from the European Commission or various platforms that compile visual material. Information about the photographer is rarely included, and the decision-making process behind the selection of photographs for publication is unknown. Data visualizations, which mostly lack captions, are, according to RARs, the sole property of Frontex, but also come from other sources. Some, for example, are produced by agencies such as the European Asylum Support Office (EASO).^1^ Controversially, a few universities have been linked to the production of some Frontex maps, but are not cited.^2^

We undertake our analysis in three stages. Firstly, following an initial screening of the material, we conduct a visually descriptive reading (Rose, 2012: 54), identifying the visual codes particular to each genre. For photographs, we pay close attention to subjects, movement, color and composition, while with maps we focus on arrows, colors, lines, legends, inscriptions, projection and orientation (Van Houtum and Bueno Lacy, 2020). Guided by our feminist postcolonial approach, we examine three overarching though interrelated motifs: 1) Subjects, e.g. migrants and border guards; 2) Practices, e.g. passport checking, fingerprinting, SAR; 3) Spaces, e.g. land, sea, air borders, as well as representations of EU/non-EU states and continents. The large dataset enables us to observe both the dominance of particular representations and if/how they change over time.

In the second stage, we operationalize gender and race as analytical lenses to conduct an intersectional visual analysis of each genre. Rather than suggesting that ‘race’ or ‘gender/sex’ are in any way physiological categories, we explore how subjects, practices and spaces are visually coded to express attributes associated with perceived hierarchical differences between men/women, (non-)Europeanness and (non-)whiteness. Committed to an intersectional approach, we also observe, where relevant, how gender and race intersect with characteristics such as nationality and age, social markers that were particularly prominent across the visual materials analyzed. The final step is an explicitly intervisual reading that explores the combined meanings of both genres. The overarching analysis identifies three dominant intervisual themes discussed in the following sections: the (in)visibilization of the body; the ascription and denial of agency; and the spatialization of borders as ‘frontier imaginings’ that oscillate between fortification and expansionism.

## Intervisual analysis

### The body in and out of sight

**Figure 1.**
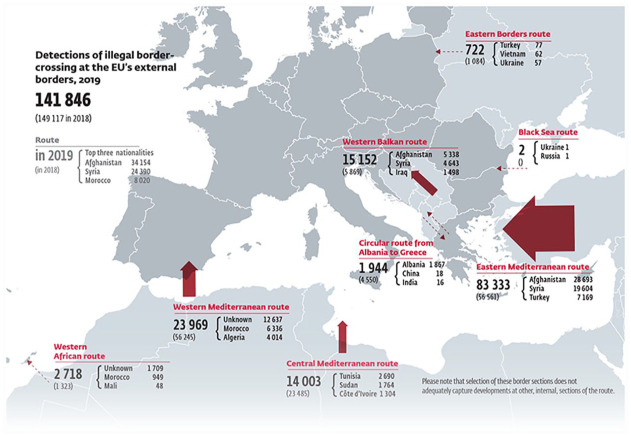
Detections of illegal border-crossing at the EU’s external borders 2015 (Frontex, 2016: 16).Source: Images © Frontex.

The RARs reproduce hierarchies between the migrant Other and European Self by (in)visibilizing bodies at different strategic points. Due to the institutional function of maps, those that feature in RARs erase the migrant body, reducing migrants to data points and their movements to lines/arrows, reclassifying migratory movements into *risks* that need to be managed. Yet they are not completely devoid of the migrant subject, but rather reinforce the trope of the ‘dangerous’ migrant established across the photographs in RARs, which mostly depict groups of largely male migrants at borders and checkpoints and on boats. These photographs are polysemic in that they could depict either a threat to Europe or individuals in need of support (Hansen, 2011; Hansen et al., 2021). However, cartographic inscriptions in accompanying maps depicting lines and arrows entering Europe ensure that the photographs are firmly anchored in the idea that the migrant constitutes a ‘threat’ that needs to be contained (Figure 1).

This hegemonic narrative about migration as one-way, illegal and always in the direction of Europe trumps an alternative reading of migration as ‘normal’, legal and often necessary for survival (Van Houtum and Bueno Lacy, 2020). Furthermore, the narrative is constituted intervisually through patterns of gendering and racialization whereby maps ascribe certain nationalities, mostly from postcolonial states, to threatening, unidirectional arrows, while photographs depict migrants as largely Black and Brown men. This is further reinforced by the order of visuals. Photographs of migrant masses and smaller groups of migrants, sometimes pictured alongside mostly white, male border guards in various humanitarian settings, are usually the first visuals included in RARs. This allows the reader to ‘picture’ the migrant subject before maps document their problematic journeys. Maps effectively zoom out, offering a bird’s-eye view of what the photographs depict, geographically locating the threat. Indeed, these maps are embedded in wider migration discourses that erase Europe’s history of empire and gendered and racialized violence.

While the Black and Brown migrant subject is translated into lines and arrows, the abstract, cartographic white Self is reproduced as the owner, maker and distributor of maps over which Black/Brown bodies become sites of intervention. The white subject is therefore not only made visible and legible through embodied and physical representations in photographs of, for example, the border guard, but also through cartographic inscriptions wherein ‘the space and place of the white human is established [. . .] through a signification system composed of text, grid lines, and logocentric and geometric symbols that establish subjectivities with cartographic authority’ (King, 2019: 87). This is supported in photographs representing border guards and/or Frontex officials poring over maps as well as photographs depicting maps appearing on surveillance screens in the so-called Frontex Situation Room (FSC).^3^ In these representations, the FSC appears like a ‘war room’, with multiple screens displaying surveillance maps being deliberated over by mostly male Frontex staff.

**Figure 2.**
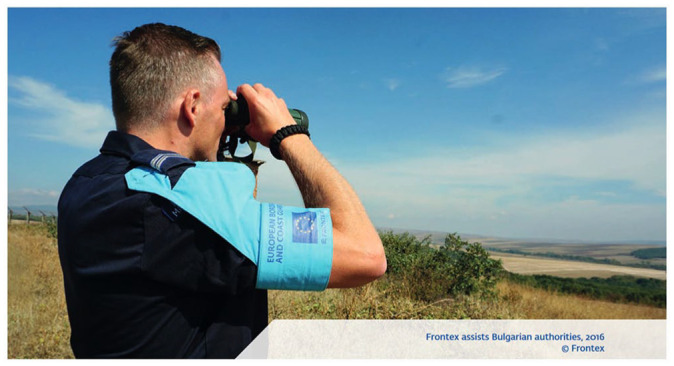
Frontex assists Bulgarian authorities (Frontex, 2017: 6).Source: Images © Frontex.

Nearly all border guards in photographs are white men who have stern facial features, wear uniforms or hi-vis jackets, and stand in strength-signifying postures. In contrast to the managerial language of RARs (Stachowitsch and Sachseder, 2019), military connotations are visually evident through the attire, equipment and practices depicted, and solidify the masculine image of patrolling, policing and protecting the border. Another significant, related visual motif that features in every RAR after 2012 depicts the male border guard, usually with his back to the camera, either gesturing towards or looking into the distance of mostly rugged landscapes or the open sea using binoculars or sometimes telescopes (e.g. Figure 2). They appear to foresee and control a potentially threatening future, establishing a contrast between unseen migrant bodies, but anticipated in the arrows on maps, and white militarized masculinities (e.g. Duncanson, 2009), which resonates with risk analysis as a preemptive practice (Tazzioli, 2018).

**Figure 3.**
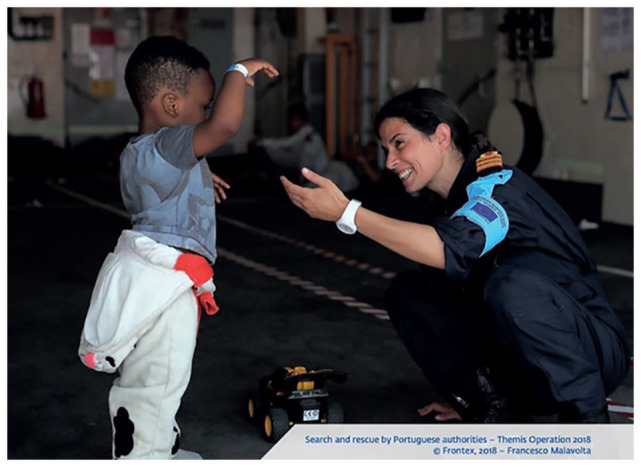
Search and rescue by Portuguese authorities – Themis Operation 2018 (Frontex, 2019: 31).Source: Images © Frontex (Francesco Malavolta).

While the representation of the white European border guard is largely represented through visuals linked to traits associated with military masculinities, it is also sustained by the representation of female border guards that link femininity to humanitarian border practices, notwithstanding that women are underrepresented in RARs. These humanitarian representations do not provide a simple counter-narrative but rather complement and sustain themes of masculinity, militarism and whiteness. Indeed, aside from occasional photographs of female guards assisting male colleagues, they are only introduced as a standalone subject from 2016 onwards. From that point, they are mostly featured in stereotypical caring roles within humanitarian contexts (e.g. Figure 3). This photograph depicts a female border guard playing with a young, Black boy around the age of 3 or 4. It is clear that she is working for Frontex due to the armband visible on her left arm. She is crouched down in front of the child and holds her hand out towards him seemingly in an act to encourage play. She is smiling joyfully in the boy’s direction, while his face is obscured by his hand. Between them is a toy truck. This representation appears to convey the ‘softer’ side of border patrolling and policing by establishing a link between women and motherhood/caregiving, yet still maintaining a binary between white saviors and racialized migrants. In light of recurring accusations of human rights violations, this visual shift is significant as it appears to challenge negative perceptions of the agency.

A second, noticeable visual shift regarding the female border guard appears in the 2021 RAR (Frontex, 2021), which is the first time a female Frontex officer is placed on the front cover as the main subject and neither in an assisting nor humanitarian role. Rather, she stands in the foreground on a boat alongside a male Frontex officer, both looking into the distance. This image aligns with the agency’s attempts to present itself as progressive with regards to gender equality, as evidenced in their 2021 campaign for International Women’s Day (IWD) which featured female border guards and used the hashtag #SecurityHasNoGender (Achilleos-Sarll et al., 2020).

Over time, and arguably in line with attempts to humanize the agency, the RARs increasingly personalize the organization by foregrounding Frontex as a key subject and brand in photographs. The shift coincides with mandate extensions that upgraded Frontex’s role from being a solely coordinating body among member states towards an independent actor within the EU (Perkowski, 2021). As mentioned, in earlier RARs, the threatening migrant mass is visually dominant, while Frontex is depersonalized, existing solely as the author of the reports, and thus the extractor and producer of knowledge about migrants/migration. However, from the 2016 RAR onwards there is a visual shift towards Frontex representatives at the border, at the Warsaw headquarters and in the FSC, recognizable as Frontex officials due to the distinctive armband. This gives the impression of a collective and centralized European effort to manage migration which transcends member state identities and conjures up associations of peacekeeping and non-aggression, with the blue Frontex armband reminiscent of the blue helmets symbolic of UN peacekeeping missions. In addition, this personalization is evident by the inclusion of the headshot photograph of the then Executive Director, Fabrice Leggeri, in the front sections, representing the physical embodiment of Frontex. Overall, correctional discourses of risk management are therefore in part reproduced through intervisually writing the body and associated subjects in and out of the RARs at different strategic points in ways that legitimize the agency and its excessively restrictive border practices.

### Practices and the ambiguity of agency

Turning from the question of who is represented to the practices different subjects engage in, we find that the RARs construct gendered and racialized meanings about migrants, migration and European border security by intervisually ascribing either ‘positive’ or ‘negative’ forms of agency. Two dominant forms of agency, ‘action *on* and *by* migrants’ (Chouliaraki and Stolić, 2019: 317) are prevalent and are co-constituted through the various practices depicted across both visual genres. Mostly set within humanitarian or technocratic contexts, border guards and Frontex representatives are usually placed in active roles, engaged in security practices such as surveillance, interceptions, processing migrants and SAR, effectively valorizing ‘action *on*’ migrants. Conversely, ‘action *by*’ migrants is either criminalized or invisibilized. Depicted as the subject of various institutional procedures such as interviewing, the migrant is rendered ‘passive’. They are represented as being ‘acted upon and affected by European border actors with a view to being identified, categorized, encamped, and re-routed’ (Chouliaraki and Stolić, 2019: 315). Photographs largely strip migrants of their agency, while data visualizations reinforce negative forms of agency by associating migratory movements with various criminal activities such as the illicit transfer of goods (as shown in photographs of smuggled products, Frontex, 2013: 54) as well as representations of ‘illegal’ border crossings. These essentialist and reductionist portrayals work to both masculinize and racialize mostly young (male) migrants, masking that the decision to migrate and/or flee violence is already an agentic act of resistance, defiance and preservation.

The 2012 RAR features a photograph of three young, muscular men putting on diving suits, and is an example of how negative forms of agency are ascribed to male migrants. The caption explains that these are Moroccans preparing to swim to Ceuta which is described as ‘a method of illegal entry’ (Frontex, 2012: 24). Maps further identify movement as illegal by criminalizing and labelling migrants as ‘illegal border-crossers’, ‘illegal over/stayers’, ‘illegal entries’ and ‘refusals of entry’, which are often linked to specific nationalities or countries of origin, either in the maps themselves or in separate graphs and charts. Through migrant encounters with border guards/Frontex officials (either physically in photographs or through cartographic inscriptions), both genres work together to establish these two (seemingly contradictory) constructions of migrant agency.

**Figure 4.**
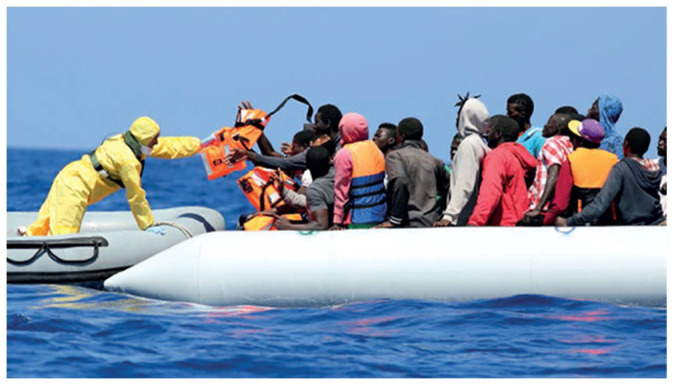
Frontex, 2016: 7.Source: Images © Frontex.

Conversely, photographs profile the agency by depicting (mostly male) border guards often operating vehicles and technological devices, linking them to sectors such as the military, surveillance and technology. Photographs show men flying planes (Frontex, 2015: 9), driving cars off-road and, in one report, driving a snowmobile (Frontex, 2019: 10) or navigating boats through the open sea. These representations assign agentic value to masculinized traits such as technological superiority, professionalism, adventurism and physical strength. The agency of the border guard is thus associated with ‘risk-taking’ while the migrant is visually constructed as ‘risky’. Representations of humanitarian practices, such as SAR, further attribute agency to border guards and their ‘acting *on*’ migrants by visually invoking white saviorism and masculinist protectionism. The cover photograph of the 2014 RAR (Frontex, 2014), for example, depicts two male border guards assisting a female migrant onto a boat, their hands covered in protective gloves. Here, the guards constitute the active subject, while the migrant is rendered passive, yet simultaneously portrayed as potentially dangerous if in direct contact. This is supported by several other photographs of border guards in clinical waterproof uniforms and face masks (Figure 4). This motif, established before the start of the COVID-19 pandemic, further underscores the active/passive binary of being both ‘*a* risk’/‘*at* risk’ (Aradau, 2004).

**Figure 5.**
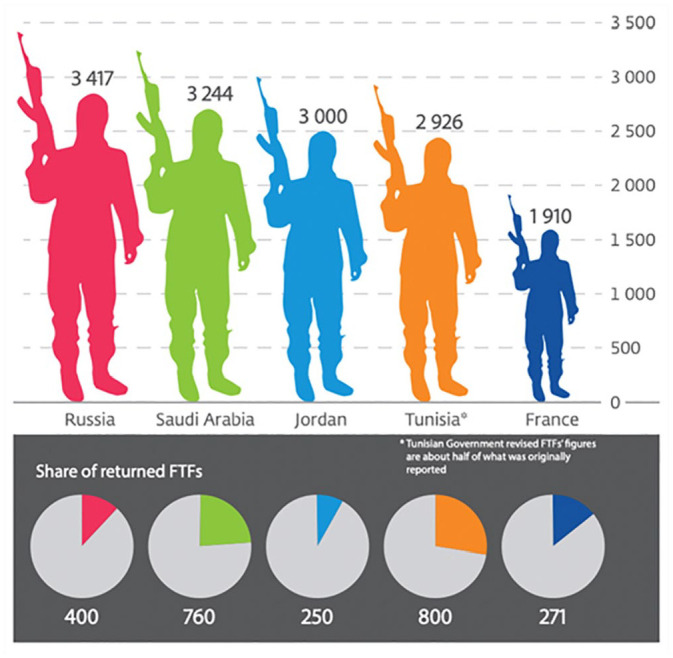
Top five countries of origin of foreign terrorist fighters who joined the conflict zones in Syria and Iraq (Frontex, 2018: 30).Source: Images © The Soufan Center.

Recently published RARs increasingly associate migrant activities with terrorism, highlighting a ramping-up of racializing discourses that equate migrants with a specific form of ‘deviant’ agency. The 2018 RAR (Frontex, 2018: 30), for example, features a graphic and series of pie charts (Figure 5) that depict ‘foreign fighters’, and is accompanied by the caption: ‘top five countries of origin of foreign terrorist fighters who joined the conflict zones in Syria and Iraq’. The graphic represents migrants as threatening through brightly coloured silhouettes of what appears to be a man pointing a machine gun upwards. This figure is replicated five times in different colours for each country of origin (Russia, Saudi Arabia, Jordan, Tunisia and France), and, being proportional to the number of fighters joining conflict zones, varies in size. The silhouette of putatively male bodies translates the ‘foreign terrorist fighter’ into an all-encompassing ‘non-European male’ that threatens Europe, while the pie charts establish a link between terrorist activities and several, mostly non-European nationalities by quantifying numbers of returns. This motif reappears in photographs, for example in the RAR 2020 (Frontex, 2020), which features a photo of three men holding automatic weapons, their silhouettes akin to those in Figure 5 (Frontex, 2020: 44). The photograph has no caption but is placed above the heading ‘Managing and interdicting terrorist mobility’ establishing a link not only between terrorism, migration and illegality, but also between terrorism, Islamism and Jihadism, thereby additionally racializing the problem of terrorism. The copyright references iStock and dates to 2010, so does not relate either to the reporting year or presumably even to the topic of migration. Yet, it is one of the very few pictures that visualizes ‘the migrant’ in this report. While the photograph presents a stylized image of this masculinized, faceless, clearly ‘foreign’ threat, the graphic supports this narrative through the use of statistics. Intervisually, this creates the impression that harm in the form of violence and terrorism is coming to Europe through ‘excessive’ levels of migration and that this threat emanates largely from young males and exclusively from outside Europe, mostly from Middle Eastern or African countries.

The intervisual analysis of depicted practices reveals a complex relationship between representations and binaries of active/passive and positive/negative forms of agency. The intersection of gendering and racialization intervisually constructs the migrant Other and European Self by rendering migrants agential in maps (though in negative, dehumanizing ways), presenting them as invading or overrunning Europe. A reversal occurs in photographs where the border guards, straddling both humanitarian and technocratic roles, are the agential actors who intervene and ‘act on’ the migrant (Chouliaraki and Stolić, 2019). A distinction is thus drawn between those who supposedly act in defence of Europe and those who can only ever be either agents of ‘threat’ or ‘helpless’ victims. There are however notable visual omissions that could pluralize the reductive and binary agency visually attached to migrant subjects described above. For example, humanizing images of the male migrant (e.g. potentially men with children) do not feature (see also Achilleos-Sarll, 2020, 2023). Moreover, the numbers of forced returns that are frequently highlighted through figures accompanying maps as well as similarly repressive bordering practices never feature in photographs.

### Borders as ‘frontier imaginings’

The gendered and racialized representation of subjects and their practices in the RARs is intimately connected to spatial imaginings that demarcate EU from non-EU territory, the latter portrayed as hostile and threatening. Subjects and spaces are interlinked through the intervisual construction of borders as ‘frontier imaginings’ (Prout and Howitt, 2009), where oscillating representations of fortification and expansionism cohere to form a hegemonic spatial narrative. In photographs, borders and surrounding landscapes are visualized as checkpoints, airports or land- and seascapes (Frontex, 2011). Maps visualize the border and (non-)European space by separating nation-states through borderlines upon which migratory movements are superimposed as arrows, circles or squares that vary in size according to the differing levels of ‘risk’ associated with them. These representations constitute a shared narrative that visualizes the European continent as feminized and threatened in relation to the racialized, non-European Other, with guards authorized to police and protect EU borders.

**Figure 6.**
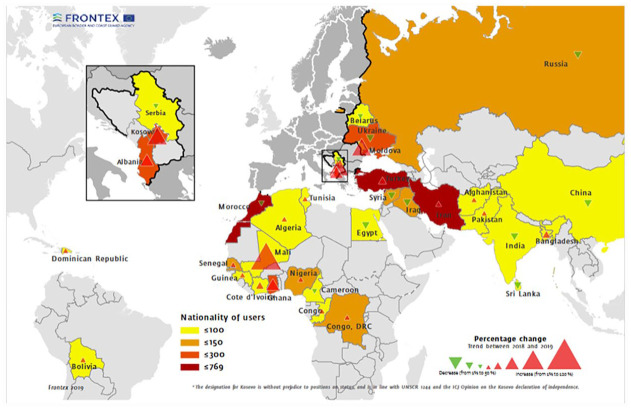
Nationality of users of fraudulent documents detected by – absolute number in 2019 and Member States relative to 2018 (Frontex, 2020: 29).Source: Images © Frontex.

In maps, this spatial separation is largely achieved through cartographic inscriptions, which include the choice of color, symbols and the direction of arrows. Various color schemes depict EU countries in blues, greens or greys, associated with neutrality, innocence and peace (Van Houtum and Bueno Lacy, 2020: 203), countries of origin and transit in light orange or red, and non-EU countries in brighter or darker colours (Figure 6), signifying abnormality and danger deviating from literal and symbolic whiteness. In earlier RARs, several maps feature levels of irregular immigration or border crossings with widening circles, bubbles or squares printed over countries of destination, sometimes almost entirely covering them. This creates the visual impression that ‘irregular’ and ‘illegal’ activity occurs within European territory (rather than at the EU’s borders), is taking over whole countries and expanding from within. Over the years, but especially after 2015, the use of large arrows overriding borders becomes more common. Referred to by Van Houtum and Bueno Lacy (2020) as ‘invasion maps’, these visuals make European territory and borders appear passive and threatened by drawing on colonial imaginaries that mark migrants as ‘unnatural intruders’ who need to be monitored and deterred (2020: 200).

Yet, the militaristic theme of (one-directional) ‘invasion’ is not the only spatial representation in RAR maps. In the context of increasing EU border externalization through the discussed mandate extensions, spatial imaginings in RAR maps have become increasingly ‘expansionist’. This perspective incorporates non-EU territories into Frontex’s remit of border management. From rather static cartographic depictions of the European continent in the earlier reports, successive maps increasingly zoom out to depict larger segments of the world, including parts of Asia and Africa, while dwarfing Europe in comparison (Figure 6). Inscriptions (squares, bubbles, circles) visualizing ‘illegal’ or ‘irregular’ activities at the border or within the EU, are increasingly placed over countries of origin and thereby ‘relocated’ to non-EU countries. Third countries, particularly postcolonial states, are represented as hosting threats waiting to come to Europe. This trend away from a visually ‘defensive’ perspective to one that is visually ‘offensive’ mirrors the de facto extension of Frontex’s sphere of influence through border externalization visualized as a potential ‘solution’ to migration ‘crises’.

The competing, though interlinked, narratives of fortification on the one hand and expansionism on the other are bridged by the intervisual construction of borders as ‘frontier imaginings’ that oscillate between a defendable line and an ambiguous and liminal space that represents risk, yet also opportunity and adventure. Constituting EU borders as incorporating zonal and linear attributes, e.g. representing them as either vast landscapes in photographs or concrete lines in maps, these imaginings enable the visual construction of borders as ambiguous spaces of both danger and potential that sustain white and masculine ‘cartographic subjectivities’ as fully human (King, 2019: 84). In the context of the EU’s own colonial past, ‘frontier imaginings’ are mobilized as the backdrop for European identity formation, bringing together the imperative of fortifying against racialized people ‘entering’ EU territory as well as the need to expand outwards.

The RARs reproduce themes of the frontier as an unknown, movable and embodied space of postcolonial anxiety by intervisually combining the changing cartographic representations described above with increasingly dramatized photographic representations of borders as threatening but also attractive spaces to be tamed by white men. Photographs stabilize the ‘white cartographic self’ (King, 2019: 87) by representing borders as the backdrop for the construction of white, European (militarized) masculinities. This can be observed through a noteworthy visual shift from relatively everyday and mundane depictions of airports and checkpoints, indicating ‘normal’ travel in earlier reports, towards more dynamic, visually striking and ambiguous views of the sea or rugged mountainous landscapes. The 2015 RAR cover photo exemplifies this trend. It is visually dramatic not only in terms of what is depicted – the bow of a ship and an angry, foamy, wavy sea, which it appears to resist – but also the way it has been presumably photoshopped to enhance the blue/turquoise color of the sea. In addition, typical frontier-like representations of the land border appear in successive reports, often combined with the motif of white, male border guards gazing out onto the landscape, which we mentioned in relation to the representation of subjects (Figure 2). Quite different from ‘fixed’ borders in maps, these photographs convey a sense of adventure, openness and uncontained vastness, which intersects with masculinized themes of adventurism and colonial and military themes of discovering unknown frontiers. At the same time, migrants are invisibilized in these frontier imaginings. Together, photographs and maps intervisually reinforce civilizational ideas of European superiority, envisioning borders not only as a demarcation that requires military protection and fortification, but which also requires preemptive, outward-looking technologies, knowledges and externalization practices. Frontex not only appears therefore as the white, masculine protector of Europe, but as a masculinized ‘pioneer’ venturing beyond EU territory to discover, manage and tame the racialized Other.

## Conclusion

Through an intervisual analysis of Frontex’s RARs (2010–2021), this article has examined the visual politics of EU border security in terms of the co-constitution of gender and race. While scholarship on the visual politics of Frontex has examined its surveillance/mapping technologies (Tazzioli, 2018; Van Houtum and Bueno Lacy, 2020) and, in one study, analysed the photographs used in its reports (Silberstein, 2021), these have not been studied together. Turning towards the visual politics of Frontex’s risk analysis, we proposed an alternative visual mapping of this increasingly central and deeply political security practice that relies on and is legitimized through the production of both photographs and data visualizations. To this end, we developed a feminist postcolonial theoretical and methodological framework to study intervisuality, presenting the first comprehensive visual examination of Frontex’s risk analysis. The article has made several theoretical, methodological and empirical contributions both to the literature on Frontex and to the visual politics scholarship on border security and (EU) migration (governance).

First, the article advanced a theoretical contribution. Conceptualizing gender and race as co-constituting analytical lenses, we argued, helps move the migration and visuality scholarship beyond identifying stereotypical representations of migrants/migration towards systematically unpacking how gendered and racialized hierarchies formed by postcolonial conditions and whiteness are reproduced through different visual genres. To understand these processes, we began from the premise that visuals work through two functionalities: as an external means of communication/representation and an institutional governance tool. We demonstrated how the visual reproduction of the EU’s border regime as gendered and racialized emerges at the intersection between these visual modes and functionalities, thereby further reifying gendered and racialized power relations.

Second, the article offered a methodological contribution by developing a feminist postcolonial approach to intervisuality for examining the visual co-constitution and reproduction of gendered and racialized power relations. This approach enabled the exploration of the meaning-making processes through which photographs and data visualizations come together in the specific construction of subjects, practices and spaces characteristic of border security. We found that photographs and data visualizations (mostly migratory maps) follow separate but linked meaning-making visual logics that anchor individual motifs in broader, hegemonic visual patterns/discourses. On the one hand, data visualizations elide violent border practices through the use of allegedly neutral, rational and authoritative aesthetics that convey a sense of depoliticized objectivity. Photographs, on the other hand, draw on more immediate, emotive forms of representation that simultaneously construct ‘risky’ and ‘deserving’ migrant subjects to create a sense of emergency. Therefore, instead of reading both genres as separate visual representations, we have demonstrated how migrants, migration and borders are made meaningful intervisually and intersectionally. This reveals how different visual codes conjointly visualize the ambiguity of risk and how this ambiguity hinges on gendered and racialized representations rooted in postcolonial power relations.

Third, the article provided an empirical contribution through the analysis of a large dataset of original visual material collected from Frontex’s RARs. While the analysis highlighted a wide range of representations, three dominant findings emerged: 1) migrant subjects, EU border authorities and Frontex are (in)visibilized at different strategic points in ways that reproduce Europe as superior, white and masculine against the threatening migrant; 2) the differential ascription of agency to border guards on the one hand and migrants on the other reproduces a hierarchy and binary between active/passive and positive/negative agency; and 3) the spatialization of borders as ‘frontier imaginings’ bridges masculinized, colonial and militaristic themes of fortification and expansionism. Gender and race function as intersectional meaning-making categories which visually place people, activities and territories within hierarchies according to their ascribed value, risk and agency. Read together, the visual politics of the RARs humanize border authorities, while dehumanizing migrant bodies and practices, denying them the capacity to act and determine their lives. Intervisually, this brings into sharp focus Self/Other imaginings that reduce the polysemic nature of visuals to hegemonic narratives around migration as one-way, migrants as racialized threats and Europe as the embodiment of softer versions of militarism and humanitarianism.

The methodological and theoretical approach we have developed could be used to study the visual politics of other actors involved in migration management and (border) security that rely on different modes of visuality. For example, the International Organization for Migration (IOM), an intergovernmental agency of the UN, also produces migratory maps and draws on photographs as a way to produce knowledge and public awareness on migration. Yet the IOM engages in a broader range of interventions with a stronger focus on humanitarian assistance and sustainable development related to, inter alia, migrant protection, assistance and healthcare, and reaches beyond Europe. Furthermore, visuals are also produced, utilized and disseminated in similar ways by more traditional security actors such nation-state militaries, the EU in the context of the CSDP, or NATO. Comparing the visual politics of these different actors would thus yield key insights into the different implications of visuals for the governance of migration and the reproduction of gendered and racialized power relations. Exploring the intervisual co-constitution of gender and race in relation to the wider discursive terrain of an organization could particularly highlight dis/continuities between humanitarian and security actors.

By taking institutions and governance structures seriously as important environments within which visuality operates, the article reveals that Frontex’s visual politics are not external to or simply a public relations activity tagged onto its migration management and policing practices, but inscribed into its very logic. As the agency continues to face massive criticism over migrant pushbacks and human rights violations (Davies et al., 2023), at the same time as member states and EU bodies continue to facilitate the agency’s expansion, the alleged neutrality and objectivity Frontex uses to police and patrol the border needs to be further scrutinized, challenged and ultimately resisted.

## Notes

1. When we include a photograph/data visualization in-text, if provided in the RAR, we also include the caption and photographer.

2. See www.michelelancione.eu/blog/2021/10/24/my-university-works-with-frontex-not-in-my-name/ (accessed 12 November 2021).

3. According to Frontex’s website, the ‘FSC has the task of providing a constantly updated picture, as near to real time as possible, of Europe’s external borders and migration situation’. See https://frontex.europa.eu/careers/who-we-are/structure/divisions/situational-awareness-and-monitoring-division/frontex-situation-centre/ (accessed 22 May 2023).
